# Is there a variance in complication types associated with ALIF approaches? A systematic review

**DOI:** 10.1007/s00701-021-05000-0

**Published:** 2021-09-21

**Authors:** Aoife Feeley, Iain Feeley, Kevin Clesham, Joseph Butler

**Affiliations:** 1 Midland Regional Hospital Tullamore, Arden Rd, Puttaghan, Tullamore, Co. Offaly R35 NY51 Ireland; 2 University College Dublin, Belfield, Dublin 4, Ireland; 3 Mater Misericordiae University Hospital, Eccles St, Dublin 7, D07 R2WY Ireland; 4 National Orthopaedic Hospital Cappagh, Cappagh Rd, Cappoge, Dublin 11, D11 EV29 Ireland

**Keywords:** Anterior lumbar interbody fusion, Approach, Complications, Outcomes

## Abstract

**Purpose:**

Anterior lumbar interbody fusion (ALIF) is a well-established alternative to posterior-based interbody fusion techniques, with approach variations, such as retroperitoneal, transperitoneal, open, and laparoscopic well described. Variable rates of complications for each approach have been enumerated in the literature. The purpose of this study was to elucidate the comparative rates of complications across approach type.

**Methods:**

A systematic review of search databases PubMed, Google Scholar, and OVID Medline was made to identify studies related to complication-associated ALIF. PRISMA guidelines were utilised for this review. Meta-analysis was used to compare intraoperative and postoperative complications with ALIF for each approach.

**Results:**

A total of 4575 studies were identified, with 5728 patients across 31 studies included for review following application of inclusion and exclusion criteria. Meta-analysis demonstrated the transperitoneal approach resulted in higher rates of retrograde ejaculation (RE) (*p* < 0.001; CI = 0.05–0.21) and overall rates of complications (*p* = 0.05; CI = 0.00–0.23). Rates of RE were higher at the L5/S1 intervertebral level. Rates of vessel injury were not significantly higher in either approach method (*p* = 0.89; CI =  − 0.04–0.07). Rates of visceral injury did not appear to be related to approach method. Laparoscopic approaches resulted in shorter inpatient stays (*p* = 0.01).

**Conclusion:**

Despite the transperitoneal approach being comparatively underpowered, its use appears to result in a significantly higher rate of intraoperative and postoperative complications, although confounders including use of bone morphogenetic protein (BMP) and spinal level should be considered. Laparoscopic approaches resulted in shorter hospital stays; however, its steep learning curve and longer operative time have deterred surgeons from its widespread adaptation.

## Introduction

The anterior approach to the lumbar spine affords excellent exposure, most commonly applied to the lower lumbar levels (L5/S1 and L4/5) [12]. The specific benefits of this approach at these levels include enabling greater volume of disc removal [36] with excellent preparation of the endplates prior to insertion of an interbody cage or graft [53], thereby allowing increased contact surface area for better fusion rates [74]. Lower operative blood loss and operative times have been reported when compared to posterior approaches [24], in addition to a reduced risk of adjacent segmental disease [49]. The avoidance of thecal sac manipulation inherently reduces risk of dural injury [61]. The advantages conferred by the anterior approach have made it an increasingly popular option for a variety of conditions including degenerative disc disease, spondylolisthesis, spinal deformity [23], and infection [53].

Acceleration of surgical techniques has been achieved through a myriad of avenues including preoperative planning using 3D printed patient specific dimensions, virtual reality-based simulation training, and advancements in surgical instruments used intraoperatively. The introduction of the DaVinci Robot by access surgeons in the approach to the anterior lumbar spine has yielded positive surgical outcomes [22]. A growing area of interest is current implant use, which has been the recent focus in research to enhance osteointegration to achieve better fusion rates [37]. Novel techniques in spinal fusion have recently been explored to minimise patient complications and hospital length of stay, with computer-assisted navigation used to increase accuracy of pedicle screw placement compared to freehand placement [47]. Efforts to mitigate against complications associated with the procedure have led to the development of variations on the surgical approach [63]. In particular, risk of to the great vessels is inherent in their mobilisation [57], whilst also at risk are the peritoneal visceral contents [30] and the ureter [30]. Damage to the hypogastric plexus may result in retrograde ejaculation in men [20]. Adequate positioning of interbody devices and minimising risk of device migration are also considerations in the type of anterior approach undertaken [1].

The goal of this review is to systematically analyse the literature to determine the rates of complications associated with the variations in the anterior approach in lumbar interbody fusion (ALIF) and other quantitative comparisons that exist within available evidence.

## Methods

### Search strategy

**Fig. 1:**
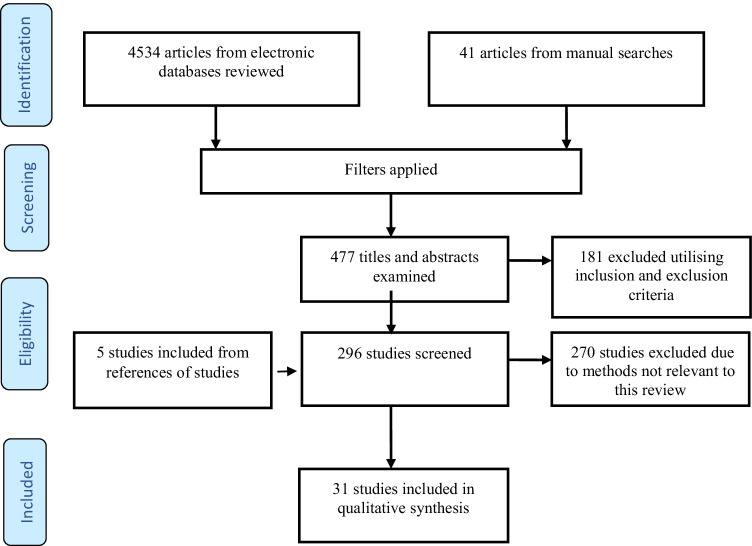
PRISMA flowchart

A systematic search in accordance with Preferred Reporting Items for Systematic reviews and Meta-Analysis (PRISMA [55]) guidelines was made of electronic databases including PubMed, OVID Medline, and Google Scholar, with study selection identification up to November 2020 (Fig. 1). Broad search terms to ensure adequate capture were used, using a combination of “Anterior Lumbar Interbody”, “ALIF”, “Complications”, “Lumbar Interbody Fusion” as keywords or MeSH search terms. This study was registered with PROSPERO under registration number: CRD42020220449.

Abstracts of relevant titles were read, with inclusion and exclusion criteria applied (Table 1), and if the inclusivity of the study was uncertain the study was read in full. Reference lists of included articles were evaluated for any additional research suitable for assessment. Data extraction was carried out independently by two reviewers (AF, IF), with 2 and 8 years specialty training completed including dedicated spinal unit residency rotations undertaken. Results were collated and presented in a systematic fashion below.

**Table 1:** Inclusion and exclusion criteria

Review criteria	Inclusion	Exclusion
Patient population	> 18, anterior interbody fusion,	< 18, oblique or lateral approaches
Study types	In vivo	Systematic reviews, case reports
Level of evidence	< IV	$\ge$ IV
Publication type	Peer reviewed, English	Abstracts, letters
Operative methods	ALIF only	Mixed approaches, disc arthroplasty, unclear methods

Study quality was assessed using the Risk of Bias Tools as per Cochrane guidelines.

Ad hoc tables were designed to summarise data from the included studies and show their key characteristics including method of fusion and use of access surgeons (Table 2) and any important questions related to the aim of this review (Tables 3 and 4). Complication rates for operative metrics, access complications, and overall complications were pooled for inter-group analysis. Meta-analysis on rates of retrograde ejaculation and vascular injuries was carried out using RevMan 5.4 using a fixed effects method to evaluate risk difference between approach groups. A difference in complication rates with a *p* value of < 0.05 was taken as significant.

**Table 2:** Studies by approach method

	Laparoscopic/other	Open
Transperitoneal	2	0
Retroperitoneal	3	16
Mixed methods	10

**Table 3:** Studies with a retroperitoneal approach

Author	Study type	Operative indications	Approach	Operation type	Device used	Level implemented	Access surgeon used
Boos et al. (2001) [9]	Prospective cohort study *N*: 20 M age:49 M:F 17:3	Discogenic back pain (*n*: 15) Spondylolisthesis *n*: 3 Non-union *n*: 2	Retroperitoneal Position: supine	Endoscopic and mini laparotomy	Syn-cage *n*: 5 Allograft ring (*n*: 15) Posterior facet joint fusion (*n*: 20)	Single level *n*: 6 Two level *n*: 14 L3/L5 L4/S1	Y
Amaral et al. (2017) [1]	Retrospective study *N*: 87 M age: 44 M:F 37:50	Degenerative disc disease Spondylolisthesis (G1)	Retroperitoneal Position: supine	Minimally invasive	NA	L5/S1 *n*: 81 L4/L5 *n*: 6	N
Malham et al. (2019) [44]	Prospective cohort study *N*: 30 M age: 58 M:F 8:22	Degenerative disc disease Spondylolisthesis (G1)	Anterolateral retroperitoneal Position: lateral decubitus	Mini-open	Integrated plate cages Polyetheretherketone ALIF cages + titanium buttress plates	Single level L5/S1 *n*: 13 Two level L4-S1 *n*: 12 Three level L3-S1 *n*: 3	Y
Bassani et al. (2018) [6]	Prospective cohort study *N*: 97 M age: 48 M:F 47:50	Degenerative disc disease (*n*: 43) Spondylolisthesis (*n*: 8) Post laminectomy syndrome (*n*: 9) Revision (*n*: 30) Degenerative scoliosis *n*: 4	Retroperitoneal Position: supine	“Keyhole” anterior skin and subcutaneous approach	Titanium cage with hydroxyapatite substitute + 3-screw plate (L5/S1), 4-screw (L4/L5), or buttress screw in posterior fixation	Single level *n*: 42 Two level *n*: 50 Three level *n*: 5	N
Mobbs et al. (2016) [52]	Prospective cohort study *N*: 227 M age: 57 M:F 108:122	Degenerative disc disease Spondylolisthesis Radiculopathy Failed fusion Scoliosis Adjacent disease	Retroperitoneal Position: NA	Mini-Pfannenstiel + midline	Stand-alone cage with bone graft	L2/L3 *n*: 3 L3/L4 *n*: 16 L4/L5 *n*: 115 L5/S1 *n*: 197	Y
Mobbs et al. (2016) [51]	Prospective cohort study *N*: 15 M age: 58 M:F 9:6	Degenerative disc disease Radiculopathy	Retroperitoneal Position: NA	Mini-Pfannenstiel + midline	Titanium/PEEK ALIF cage + allograft supercritical CO_2_ + BMP Percutaneous pedicle fixation *n*: 2	Single level *n*: 11 Two level *n*: 3 Three level *n*: 1	Y
Hironaka et al. (2013) [29]	Retrospective cohort study *N*: 142 M age: 64.3 M:F 82:60	Degenerative disc disease Intervertebral foraminal stenosis Disc height loss Spondylolisthesis	Retroperitoneal Position: lateral decubitus	Mini-open	BAK *n*: 25 Stabilis *n*: 5 L-varlock *n*: 112 Apacerum powder and autologous bone graft	Single level L4/L5 *n*: 142	Y
Comer et al. (2012) [20]	Retrospective consecutive cohort study *N*: 472 M age: 42 M:F 472:0	Spondylolisthesis Degenerative disc disease	Retroperitoneal Position: NA	Open Transrectus *n*: 297 Anterolateral *n*: 75	FRA Mesh cage ICBG Demineralised bone graft + buttress screw: 418	Single level L5/S1 *n*: 302 Two level L4/S1 *n*: 170	N
Garg et al. (2010) [27]	Retrospective cohort study *N*: 212 M age: 53.8 M:F 92:120	Degenerative disc disease Spondylolisthesis	Retroperitoneal Position: NA	Mini-Pfannenstiel	NA	Single level L4/L5 *n*: 22 L5/S1 *n*: 149 Two level L4/S1 *n*: 41	Y
Siepe et al. (2015) [66]	Prospective cohort study *N*: 71 M age: 47 M:F 26:45	Degenerative disc disease	Retroperitoneal Position: NA	Mini-open	Syn-fix LR BMP *n*: 54 Autologous bone graft *n*: 11 Demineralised bone *n*: 5	Single level L5/S1 *n*: 71	N
Aunoble et al. (2006) [3]	Prospective cohort study *N*: 20 M age: 39 M:F 9:11	Spondylolisthesis (G0/1)	Retroperitoneal Position: supine	Video assisted	Union cage *n*: 15 Lordotec *n*: 5 Pyramid anterior plate *n*: 20	Single level L5/S1 *n*: 20	N
Manunga et al. (2020) [46]	Retrospective cohort study *N*: 1178 M age: 54 M:F 514:664	NA	Retroperitoneal Position: NA	Midline	NA	Single level *n*: 422 Two level *n*: 450 Three level *n*: 208 Four level *n*: 93 > Five level *n*: 8	Y
Lucas et al. (2016) [42]	Retrospective cohort study *N*: 84 M age: 47 M:F 37:47	Herniation *n*: 30 Post laminectomy instability *n*: 8 Discogenic back pain *n*: 46	Retroperitoneal Position: lithotomy	Open	Osteoinductive protein + posterior approach *n*: 43	Single level *n*: 54 Two level *n*: 25 Three level *n*: 1	Y
Brau (2002) [10]	Retrospective cohort study *N*: 687 M age: 18–79 M:F 345:339	NA	Retroperitoneal Position: supine	Mini-open	NA	Single level L3/4 *n*: 23 L4/5 *n*: 127 L5/S1 *n*: 242 Two level L4/S1 *n*: 194 L3/5 *n*: 31 Three level *n*: 35 Four level *n*: 4	Y
Kapustka et al. (2020) [36]	Retrospective cohort study *N*: 51 M age: 47 M:F 27:24	Degenerative disc disease Radiculopathy Foraminal stenosis Disc herniation	Retroperitoneal Position: NA	Minimally invasive	Syn-Fix PEEK	Single level *n*: L5/S1	Y
Mamuti et al.(2016) [45]	Retrospective cohort study *N*: 35 M age: 52.8 M:F 10:25	Failed posterior approach	Retroperitoneal Position: supine	Mini-open	NA	Single level L4.L5 *n*: 14 L5/S1 *n*: 15 Two level L4/S1 *n*: 6	N
Brewster et al. (2008) [11]	Retrospective comparative study *N*: 128 M age: 41 M:F 95:33	NA	Retroperitoneal Position: NA	Open	Iliac crest bone graft Bioprosthetic	Single level L4/L5 L5/S1 Two level L4/S1	Y
Vazquez and Gireesan (2003) [69]	Retrospective cohort study *N*: 46 M age: 40 M:F 34:12	Degenerative disc disease Spondylolisthesis Disc infection Retro spondylolisthesis	Retroperitoneal Position: supine	BERG	BAK cage *n*: 33 Femoral ring *n*: 8 Titanium mesh *n*: 3 Brantigan cage *n*: 1 + Posterior pedicle screw *n*: 12	Single level Two level	Y
Carragee et al. (2011) [16]	Retrospective comparative study *N*: 69/174 M age: 42.9/40.9 M:F NA	Disc herniation Spondylosis Spondylolisthesis	Retroperitoneal Position: NA	Open	rhBMP-2 *n*: 69 Demineralised bone matrix/iliac crest bone graft *n*: 140 FRA *n*: 172 Cage mesh *n*: 2 Local autograft *n*: 15	Single level L4/L5 L5/S1 *n*: 155 Two level L4/S1 *n*: 88	N
Lubelski et al. (2013) [41]	Retrospective comparative study *N*: 110 M age: 51.1 M:F 110:0	Degenerative disc disease Spondylolisthesis Pseudoarthrosis Osteomyelitis Fracture	Retroperitoneal Position: NA	Open	BMP	Two level L4/S1	N

**Table 4:** Retroperitoneal and mixed methods studies

Sasso et al. (2003) [65]	Prospective comparative study *N*: 146 M:F 146:0	Degenerative disc disease	Retroperitoneal *n*: 116 Transperitoneal *n*: 30 Position: supine	Open	rhBMP-2 (*n*: 78) Iliac crest bone graft (*n*: 68)	One level L4/L5 or L5/S1	Y
Kaiser et al. (2002) [35]	Retrospective comparative study *N*: 98 M age: 43 M:F 43:55	DDD spondylolisthesis (G1) Pseudoarthrosis multiple diagnoses	Transperitoneal [35, 48] Position: NA	Laparoscopic *n*: 47 Mini-open *n*: 51	NA	Single level *n*: 88 L5/S1 *n*: 81 L4/L5 *n*: 7 Two level *n*: 10 L4/S1 *n*: 9 L3/L5 *n*: 1	Y
Frantzides et al. (2006) [26]	Retrospective cohort study *N*: 28 M age: 43 M:F 15:13	Degenerative disc disease spondylolisthesis post laminectomy syndromes	Transperitoneal Position: Trendelenburg	Laparoscopic	BAK RAY Lordotic LT cages Iliac bone graft + BMP Posterior pedicle screw *n*: 10	Single level L5/S1 *n*: 28	Y
Escobar et al. (2003) [25]	Retrospective comparative study *N*: 135 M age: 43 M:F 50:85	Degenerative disc disease Spondylolisthesis Previous fusion pseudoarthrosis	Retroperitoneal vídeo assisted *n*: 30 Transperitoneal insufflation *n*: 34 Position: NA	Retroperitoneal: open (G4) (*n*: 20) Video assisted (G2) (*n*: 30) Transperitoneal insufflation (G1) (*n*: 34) Mini-lap extraperitoneal (G3) (*n*: 30)	Cylindrical cage Bone grafting	NA	Y
Cowles et al. (2000) [21]	Retrospective comparative study *N*: 75 M age: 45/41 M:F 33:43	Degenerative disc disease Failed previous surgery	Transperitoneal Position: Trendelenburg	Laparoscopic *n*: 55 + open *n*: 20	NA	NA	Y
Burkus et al. (2002) [14]	RCT *N*: 279 M age: 43.3 M:F 146:133	Degenerative disc disease Spondylolisthesis	Retroperitoneal *n*: 226 + transperitoneal *n*: 53 Position: supine	Open	LT-CAGE BMP *n*: 143 Iliac crest bone graft *n*: 136	Single level L4/L5 *n*: 69 L5/S1 *n*: 109 L5/L6 *n*: 1	N
Lavelle et al. (2014) [39]	RCT *N*: 73 M age: 44 M:F 32:42	Degenerative disc disease Discogenic back pain	Retroperitoneal *n*: 17 Transperitoneal *n*: 56 Position: NA	Midline (trans) + paramedian (retro)	BAK *n*: 32 SAC *n*: 41 ICBG	Single level L4/L5 *n*: 19 L5/S/1 *n*: 37 Two level L4/S1 *n*: 17	NA
Zdeblick and David (2000) [74]	Prospective comparative study *N*: 50 M age: 40.5 M:F 24:26	Discogenic pain Pseudoarthrosis Failed surgery	Transperitoneal *n*: 25 Retroperitoneal *n*: 25 Position: NA	Laparoscopic *n*: 25 + mini-open *n*: 25	BAK Lordotec Femoral cortical Interbody fusion allograft	Single level L4/L5	Y (Lap group)
Geerdes et al. (2001) [28]	Retrospective cohort study *N*: 30 M age: 43 M:F 8:22	Discogenic back pain Radiculopathy Degenerative disc disease	Transperitoneal Position: Trendelenburg	Laparoscopy	BAK	Single level L4/L5 *n*: 3 L5/S1 *n*: 25 Two level L4/S1 *n*: 2	Y
Chung et al. (2003) [19]	Prospective comparative study *N*: 44 M age: 49.5 M:F 11:33	Discogenic back pain	Transperitoneal *n*: 22 Retroperitoneal *n*: 22 Position: NA	Mini-open *n*: 22 Laparoscopic *n*: 22	Brantigan carbon cage	Single level L5/S1	Y (Lap group)
Safaee et al. (2020) [62]	Retrospective cohort study *N*: 938 M age: 57 M:F 427:511	Spondylolisthesis Deformity Infection Trauma Tumour Pseudoarthrosis	Retroperitoneal *n*: 898 Transthoracic *n*: 40 Position: NA	Open	NA	Single level *n*: 350 Two level *n*: 396 Three level *n*: 164 > Four level *n*: 28	Y

## Results

Our search yielded 4575 publications. The 31 studies satisfying inclusion criteria included a total of 5728 patients, 5408 undergoing and ALIF via a retroperitoneal approach, 320 via a transperitoneal approach, with methods enumerated below (Table 2).

Included studies in this review consisted of two randomised control trials: 10 prospective cohort studies and 19 retrospective studies. There was a trend toward the retroperitoneal approach with all study methodologies. Bias was assessed using Cochrane recommended tools for systematic reviews, with no studies deemed high risk during assessment. Non-randomised control trials studies were assessed using ROBINS-I tool, generally demonstrating unclear risk in relation to missing data, measurement of outcomes, and bias in selection of reported results, in part due to the study methodologies of retrospective data collection.

### Complications

Simple pooling of outcomes evaluated was carried out across studies included for review to assess perioperative and postoperative outcomes between approach methods. Complications included for analysis included rates of RE, vascular injury, haematological complications including deep venous thrombosis and haematoma formation, injury to the viscera, development of Dural tears, wound infections, hernias, and the incidence of postoperative ileus. Total patients included per group were used for analysis, except rates of RE. Where no gender breakdown was available in the studies, complication and patient population were excluded, with results summarised below (Table 5).

**Table 5:** Complications by approach type

	Trans lap *n*: 241	Trans open *n*: 154	Retro another *n*: 227	Retro open *n*: 5106
Average operative time	180 min	165 min	250 min	133 min
EBL	103 ml	295 ml	204 ml	190 ml
Average length of stay	3.7 days	4.25 days	3.7 days	4.5 days
Retrograde ejaculation	22%	12.2%	2.2%	2%
Vessel damage	2.1%	0.7%	3.1%	3.03%
DVT	0.5%	NA	0.35%	1.4%
Haematoma formation	NA	NA	0.7%	0.6%
Visceral damage	2.1%	NA	0.4%	0.37%
Hernia	NA	NA	NA	1.3%
Wound infection:	NA	0.7%	0.35%	1.9%
Ileus	2.1%	3.7%	NA	5%
Dural tears	NA	0.7%	NA	0.04%

## Meta-analysis

Meta-analysis of studies directly comparing transperitoneal and retroperitoneal approaches for overall complications, rates of retrograde ejaculation (RE), and vessel injury was carried out, with 5 studies included for analysis of RE and 3 studies comparing vascular outcomes, and overall outcomes between approaches. An *I*^2^ value of 0–41% demonstrated for all comparisons, demonstrating moderate homogeneity.

### Retrograde ejaculation

**Fig. 2:**
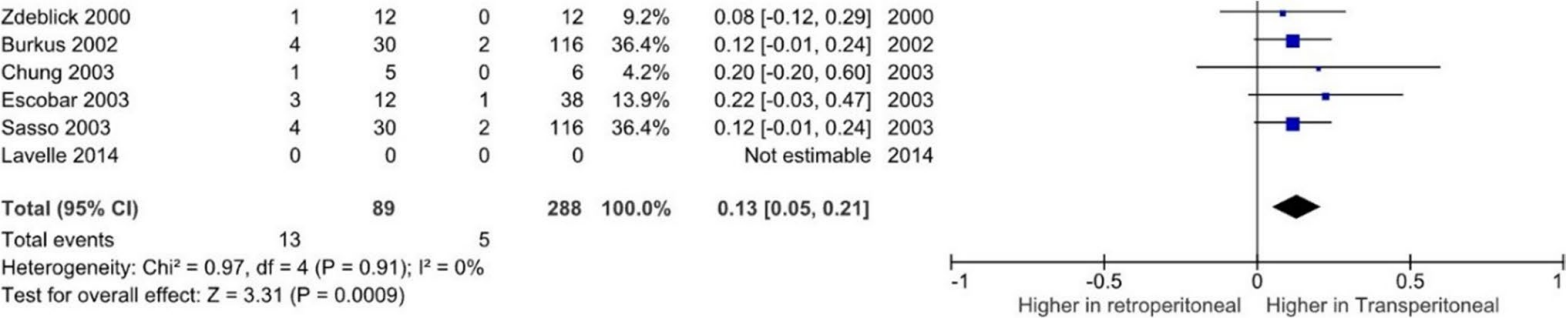
Meta-analysis demonstrating rates of retrograde ejaculation between approaches

Comparative studies demonstrated a significantly higher risk of RE (Fig. 2) using the transperitoneal approach (*p* < 0.001; CI = 0.05–0.21).

### Vessel injury

**Fig. 3:**
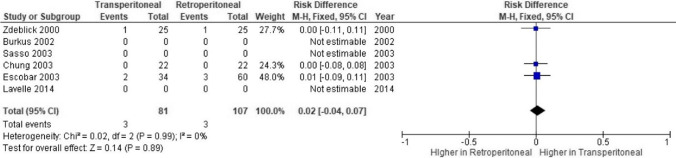
Meta-analysis demonstrating rates of vessel injury between approaches

Number of vessel complications in comparative studies had a generally low reporting (Fig. 3); however, rates of vessel injury by approach were not found to be significant (*p* = 0.89; CI =  − 0.04–0.07).

### Overall complications

**Fig. 4:**
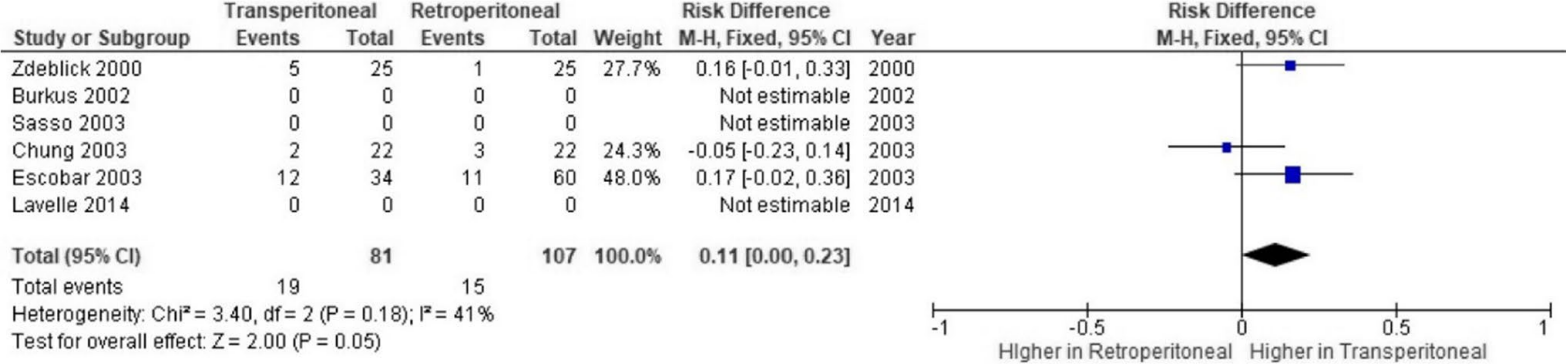
Meta-analysis demonstrating rates of overall complications between approaches

Data regarding overall complications (Fig. 4) was reported in only 3 comparative studies. The mean number of peri and postoperative complications in the retroperitoneal (*n* = 5) and transperitoneal (*n* = 6) groups, respectively, with this difference deemed not statistically significant (*p* = 0.05; CI = 0.00–0.23). The *I*^2^ value was 41%.

## Discussion

### Use of access surgeons

Anterior access to the spine is purported to provide better fusion rates; less blood loss, soft tissue injury, neural injury; and shorter operative time [5]. These benefits are weighed against the risk of vascular and visceral damage during exposure of the spine [58]. Efforts to mitigate these complications have included the use of “access surgeons” to provide safe passage. Conflicting evidence on the advantage conferred by the presence of general and vascular surgeons exists [32], a potential cause thought to be the variable training provided to spinal surgeons depending on healthcare system’s practice of access surgeon use [1]. A recent meta-analysis found use of access surgeons is associated with increased rates of retrograde ejaculation (RE) and arterial injury (*p* < 0.001), with fewer postoperative complications noted than studies without their input [59]. This was felt likely to be in part due to the use of access surgeons predominantly in multilevel exposure, or difficult anatomical variances. Three studies [2, 44, 46] evaluated the role of access surgeons in the anterior spinal surgery reported a low vascular complication rate due to the presence of the access surgeon for the entirety of the operation. In contrast, Garg et al. [27] reported a 64% vascular injury occurring on approach despite the presence of a vascular surgeon. Whilst Chiriano et al. [17] previously emphasised the importance of access surgeons primarily for continued vascular trainee exposure to open surgery, three studies in this review [21, 25, 26] found a significant learning curve associated with their laparoscopic ALIF approach despite the presence of fully qualified general surgeons, with Cowles et al. [21] finding the conversion rate dropped from nearly 50 to 0% across the study time frame. The need for continued surgical resident exposure to ALIF procedures to ensure sufficiently experienced surgeons can provide access to the spine has been emphasised, regardless of surgical specialty [11]. Previous research on the impact of access surgeons on patient outcomes did not evaluate the relationship between level of surgical training obtained and complication rates outlined [59]; however, the validity of trainees as access surgeons in this role and familiarity of the access surgeon to the procedure warrant further evaluation given the high morbidity associated with complications in both transperitoneal and retroperitoneal approaches.

The impact of access surgeons on operative time has yet to established. Jarrett et al. [32] found similar rates of complication rates in patient groups with both access surgeons and no access surgeons present but noted that access surgeons were more likely to participate in multilevel exposure procedures and should be considered when factoring the impact of access surgeons on total operative time. Mogannam et al. [54] evaluated the impact of previous abdominal surgery on patient complication rates, with no significant increase in rates noted.

### Open vs. laparoscopic approaches

Whilst advantages including shorter inpatient stays, fewer major complications, and less blood loss are associated with the laparoscopic approach [15], the risk of complications due to the anatomical variation at the level of approach, a steep learning curve, and the required knowledge of intra-abdominal anatomy [25] with longer operative times have led to widespread adoption of the open retroperitoneal approach. Cowles et al. [21] reported the highest conversion rate at 38%, with risk factors including involvement of more than one spinal level, adhesions, bleeding, and levels other than L5/S1. The most common reason for conversion reported was to gain adequate control following vessel injury [25, 35], with Zdeblick et al. [74] reporting a 16% conversion rate due to inadequate exposure laparoscopically.

Estimated blood loss was less using non-open approaches, although this did not reach significance (*p* = 0.1), with longer operative times reported (*p* = 0.1). Blood loss has been indicated to be a poor objective measurement, due to the association of hidden blood loss in anterior lumbar interbody fusion operations [34]. Average length of stay was shorter in patients undergoing a non-open approach (*p* = 0.01). Complications arising from open procedures including wound infection and ileus were likely the cause for the longer inpatient stay in this cohort.

### Patient positioning

Patient positioning is an important preoperative step to minimise anaesthetic risks, reduce risk of damage to structures during access to the spine, and the need for intraoperative repositioning [44]. A study evaluating the position of the great vessels in relation to the anterior spine found the bifurcation of the aorta was higher whilst in the prone position; the left common iliac vessels are at greater risk whilst patients are supine [7], with the lateral decubitus position thought to aid gravity mobilise abdominal contents including the great vessels away from the operative field [44]. Indeed, efforts to mitigate approach-related complications associated with ALIF have led to a rise in the use of the oblique lumbar interbody fusion (OLIF) [33], with favourable outcomes on rates of ileus compared to ALIF [72]. With the minimally invasive approach OLIF has a vascular complication rate than that of ALIF; however, further research on its long-term outcomes is required [73]. With a rise in the use of the lateral decubitus positioning with recently developed spinal instruments, morphometric evaluation of the great vessels indicates the L5/S1 window is larger in the supine position compared to that of the lateral decubitus position (*p* < 0.0001) [18]. The impact of patient positioning in the lazy lateral position offers a potential protection against risk to vascular structures, and patient outcomes demonstrated that the lazy lateral position yielded no vascular or visceral-associated patient complications [56]. Molloy et al. [56] felt the use of muscular windows during initial access contributed to the reduction in postoperative hernia rates. Reporting of patient positioning was inconsistent across the studies in this review; of those listed, supine was the most common, with three studies having patients in Trendelenburg [21, 26, 28], two in lateral decubitus [29, 44], and one in lithotomy [42], with small numbers preventing further analysis on their use.

### Vascular injury

Vascular damage is a known complication arising whilst obtaining access to the spine [38]. Conflicting reports on the impact of factors contributing to vascular injury exist, including patient co-morbidities, spinal level [17], and laparoscopic vs. open approach [71]. The transperitoneal approach significantly increased the risk of vascular damage compared to retroperitoneally in one review [71], a finding refuted in other studies [2, 31]. Risk of vessel injury was found to be higher in retroperitoneal approaches in this review, a difference not reaching significance (*p* = 0.07). Manunga et al. [46] noted their transperitoneal approach allowed them greater scope to mobilise the vessels thus aiding a reduction in damage, and greater operative control, congruent with Lucas et al. [42] finding the level of bifurcation required retraction of the iliac vessels far to the right with the retroperitoneal approach, the most common cause of vessel injury.

The ALIF approach at L4/L5 is limited by the rates of vascular injury and degree of vessel mobilisation required [73]. Research has failed to demonstrate a predictive correlation between anatomical pelvic parameters and overlying vessel location, indicating independent vascular planning may be required in the surgical approach [4]. Chiriano et al. [17] found vascular injuries occurred most commonly during the L4/L5 approach during exposure between the left iliac artery and vein resulting in a significantly higher rate injury (*p* < 0.001), echoed by two studies in this review [26, 46], with Manunga et al. [46] finding 84.6% vessel injuries occurring at the L4/L5 exposure step using an open approach, most commonly due to avulsion of the iliac vessels. Multilevel exposure was also found to increase risk of vascular injury (*p* < 0.001) [27]. Type of cage and associated vascular complications were not discussed in the studies included in this review. Vascular complications have been found to be significantly more common with the use of threaded cages compared to non-threaded cages [64]. With the development of novel 3D porous cages in ALIF [50], impact of cage type on rates of vascular injury should be considered. Vascular complications including deep venous thrombosis (DVT) and pulmonary embolism (PE) have been reported to have a higher association in ALIF operation, with additional intraoperative posterior steps increasing the risk [31]. The highest incidence in this review occurred in the retroperitoneal open approach, although variation in reporting complications was prevalent in studies included. One study found a correlation (*p* = 0.022) between DVT and multilevel exposure, and males (*p* = 0.013) [27], with 10% DVTs occurring following extensive iliac reconstruction in another [46].

### Rates of RE

Ejaculatory disorders (ED) are a commonly cited complication occurring in lumbar spinal surgeries, thought to occur primarily due to hypogastric plexus injury during access to the surgical corridor [40]. Factors associated with an increased risk of ED are obesity [8] and use of BMP. Neuroinflammation is a side effect of BMP investigated by the Food and Drug Administration (FDA), with indications that its use near the hypogastric plexus could result in complications arising from compound leakage. Burkus et al. [13] evaluated a series of anterior lumbar spine studies, finding a correlation with use of BMP and RE not reaching significance. Three studies in this review directly evaluated the effect of BMP in ALIF rates of RE [16, 20, 41], with mixed findings. Comer et al. [20] noted a found a significant association between use of BMP and rates of RE (*p*=0.0012). Carragee et al. [16] reported a similar finding (*p*=0.023), while Lubelski et al. [41] found no significant association (*p*=1). Burkus et al. [14] found a significant correlation between urinary retention and use of BMP (*p* = 0.04), a complication not commonly listed in studies evaluating its efficacy; thus, a true incidence of the relationship is unknown.

The transperitoneal approach had a significantly higher association with RE compared to other approaches in this review (*p* = 0.0009). Evaluation of available data demonstrated 92% cases RE occurred at L5/S1 in two studies with significantly high rates [28, 35]. Similarly, Sasso et al. [65] found 85% of the patients who developed RE underwent exposure at the L5–S1 level. This finding was supported by Carragee et al. [16] who suggested the rates of RE in relation to the L5/S1 junction may be in part due to the bilateral injury risk associated with dissection of the aortic bifurcation. Given the FDA supported findings regarding the risk of BMP use, findings in two of these studies [35, 65] should be taken in context of an unclear breakdown of BMP use between control and intervention groups. Many studies reported their retrospective study method precluded accurate reporting rates of RE [46, 62], which should be considered in the context of these findings.

### Visceral injury

Visceral complications including ureteric injury, enterotomy, bladder rupture have all been found with the ALIF approach [71]. Reported rates of visceral injury were low from all approaches in this review. Three studies using a retroperitoneal approach reported peritoneal rupture rates ranging from 2.29 [1] to 16% [25] with no long-term complications. Boos et al. [9] reported all peritoneal rupture cases occurred primarily during extraperitoneal port insertion, early in the data collection stages of the study, and have thus attributed this to a learning curve.

### Postoperative ileus

Rates of postoperative ileus in ALIF patients vary in the literature, with between 2 and 58% reported [60], most commonly occurring between 2 and 5.4% [43], and occurring in both transperitoneal and retroperitoneal approaches [68]. Open ALIF approaches in this review had higher rates of postoperative ileus reported compared to laparoscopic procedures, with no significant difference between transperitoneal and retroperitoneal approaches (3.54% vs. 2.60%; *p* > 0.05). Bowel obstruction is a not uncommon complication associated with ALIF, with retroperitoneal structure mobilisation thought to be associated with the incidence of pseudo-obstruction [68]. Frantzides et al. [26] had one case of small bowel obstruction which they attributed to posterior peritoneum-cage adhesion and recommended peritoneal closure to reduce complications. Manunga et al. [46] identified their bowel ischemia from SMA injury as secondary to patient aneurysm identified postoperatively, recommending careful preoperative evaluation to identify patients at increased risk of complications.

### Neurological complications

Dural tears are a complication in lumbar surgery associated with significant secondary complications including intracranial haematoma and dural fistulae [70]. Additionally, dural tears are associated with an increased risk of wound infections, neurological deficits, and perioperative systemic complications [67]. Three cases of dural tears were reported in this review, two occurring in the open retroperitoneal approach [11, 29] with an incidence rate of 0.04%, and one in the transperitoneal open group (0.7%) [35].

## Conclusion

The open retroperitoneal approach is an established method for anterior lumbar interbody fusions with an acceptable safety profile. Transperitoneal studies are underpowered, with a dearth of data available. Research indicates a significantly higher risk of RE with this method; however, confounders including use of BMP and insufficient reporting of spinal level approaches should be considered. Laparoscopic approaches have shorter inpatient stays, with less postoperative complications including wound infection and ileus; however, its steep learning curve and longer operative times deter surgeons from its widespread adaptation.

## Data Availability

Not applicable.
